# Experiments on the Hearts of the Sturgeon, Snapping Turtle, and Frog, When Removed from the Body

**Published:** 1845-07

**Authors:** F. G. Smith

**Affiliations:** Lecturer on Physiology in the Philadelphia Medical Association, &c.


*Experiments on the hearts of the Sturgeon, Snapping Turtle, and Frog, when removed from the body.* By F. G. Smith, jr., M. D., Lecturer on Physiology in the Philadelphia Medical Association, &c. (Communicated by Professor Dunglison.)

*Philadelphia*, June 20th, 1845.

Professor Dunglison,

*Dear Sir*,—On the 27th of May I had an opportunity of witnessing the long continued contraction of the heart of a sturgeon, after its removal from the body. Through the kindness of Professor Mitchell, I had the pleasure of seeing this interesting phenomenon some days before, but as I was not then able to watch it closely, I determined to repeat the experiment myself.

Having procured a strong and lively fish on the morning of the 27th, he was kept in full vigour, by repeatedly throwing water over the gills, until all the necessary arrangements had been made. In the presence of several medical friends, the heart was removed, having first taken the precaution to tie the vena cava and the pulmonary artery; by this means retaining a good deal of blood within the organ. It was found, however, that although the heart pulsated at the rate of twenty-four times in a minute, the contractions were not so vigorous as had been expected. The ligature was then removed from the pulmonary artery, and the heart allowed to empty itself by its own powers, thus showing, in an interesting manner, the propulsion of the blood from the central organ. After it had completely emptied itself, the cavities were inflated with air from the lungs,—then, on re-applying the ligature, the contractions of the heart greatly increased in force, still retaining the same number of pulsations as before. They commenced at the superior part of the auricle, and were transmitted by a rapid, wave-like motion, over the body of the auricle, until they reached the ventricle, which immediately contracted with a short, jerking movement. The time occupied by the contraction of the ventricle was shorter than that occupied by the contraction of the auricle. As the heart became dry, the vigour of the contractions diminished, but by moistening it with water, it was in some degree restored. Gradually, however, the number of pulsations was reduced,—the ventricle ceasing to contract first. As the auricle dried, the pulsations ceased in those parts that dried first, beginning at the most superior parts, and then it was interesting to observe that the order of contractions was reversed, commencing at the most dependent portion. The number of contractions slowly diminished through the day, from twenty-four in the minute to nine, which was the number at 11 o'clock in the evening. At 7 the next morning, the middle portion of the auricle (the only part that remained moist) was still contracting slightly, though, by irritating it with a pointed instrument, its contractions were very distinctly marked. At any time during the preceding day and evening, motion could be produced by irritation. From other engagements the opportunity was wanting to prolong the observation after seven; and returning at eight, the pulsations had entirely ceased. They had continued twenty-two hours after the removal of the heart from the body.

On the 19th of June I repeated the experiment, assisted by Drs. Allen and Neill. On this occasion we used, not only the heart of a sturgeon, but, in addition, that of a snapping turtle and a frog. The heart of the sturgeon was removed at 11 A.M., having taken the same precaution as before. We again observed the same occurrence, that the organ did not begin to contract forcibly till the cavities were emptied of blood and filled with air, when the movements were even more energetic than in the first experiment. They did not last, however, so long as in the first, having entirely ceased twelve hours after removal. The same phenomena of reversed action were witnessed in this case.

The heart of the frog continued to contract an hour longer than that of the sturgeon, lasting thirteen hours in all. That of the snapper is still contracting at this time, (twenty-five hours after removal,) though the space over which the motions are evident, is small, being merely the base of the auricle, which alone has remained moist. It is worthy of remark, that the auricles have participated more than the ventricles in all these experiments, in the motion of this organ.

| Heart of Sturgeon. | Frog. | Turtle. |
|---|---|---|
| 11, A. M., 32 puls. in minute.. | 1 o'clock, 60 in minute. | $1\frac{1}{2}$ o'cl'k, 20 in minute. |
| $11\frac{3}{4}$, 24 " " | 3 " 40 " | 3 " 14 " |
| 12, 18 " " | 7 " 15 " | 6 " 12 " |
| $12\frac{1}{2}$, 20 " " | 10 " 10 " | 10 " 10 " |
| 1, 20, very little motion in ventricle. | $11\frac{1}{2}$ " 10 " | $11\frac{1}{2}$ " 10, vent. slight. |
| 2, 10 ventricle just ceased. | 12 " Very slight. | |
| 3, 4 contractions of auricle in minute. | | |
| 6, 6 " " " | | |
| 7, 4 " " " | | |
| 10, 4 " " " | | |
| 11, Auricle just ceased. | | |

The ventricle ceased to contract in the night, but at six, A.M., the next morning, the auricles were still contracting, (though very dry,) at the rate of eight in the minute. At 8 o'clock, on slight irritation, eight times per minute. Those contractions still continued till 3, P. M., when they were arrested by putting the heart in warm water, with the hope of increasing them. Total time, $25\frac{3}{4}$ hours.

The heart of the sturgeon was, *perhaps*, arrested by its becoming emphysematous, from having too much air forced into it in the first instance.

Yours, respectfully,

F. G. Smith, Jr.

